# The identification of Oligo-Miocene mammalian palaeocommunities from the Riversleigh World Heritage Area, Australia and an appraisal of palaeoecological techniques

**DOI:** 10.7717/peerj.3511

**Published:** 2017-06-30

**Authors:** Troy J. Myers, Karen H. Black, Michael Archer, Suzanne J. Hand

**Affiliations:** Palaeontology, Geobiology and Earth Archives (PANGEA) Research Centre, School of Biological, Earth and Environmental Sciences, University of New South Wales, Sydney, NSW, Australia

**Keywords:** Oligocene, Miocene, Palaeoecology, Palaeocommunity, Riversleigh, Local faunas, Mammal, Classification, Ordination, Multivariate

## Abstract

Fourteen of the best sampled Oligo-Miocene local faunas from the Riversleigh World Heritage Area, north-western Queensland, Australia are analysed using classification and ordination techniques to identify potential mammalian palaeocommunities and palaeocommunity *types*. Abundance data for these faunas are used, for the first time, in conjunction with presence/absence data. An early Miocene Faunal Zone B and two middle Miocene Faunal Zone C palaeocommunities are recognised, as well as one palaeocommunity type. Change in palaeocommunity structure, between the early Miocene and middle Miocene, may be the result of significant climate change during the Miocene Carbon Isotope Excursion. The complexes of local faunas identified will allow researchers to use novel palaeocommunities in future analyses of Riversleigh’s fossil faunas. The utility of some palaeoecological multivariate indices and techniques is examined. The Dice index is found to outperform other binary similarity/distance coefficients, while the UPGMA algorithm is more useful than neighbour joining. Evidence is equivocal for the usefulness of presence/absence data compared to abundance.

## Introduction

**Figure 1:**
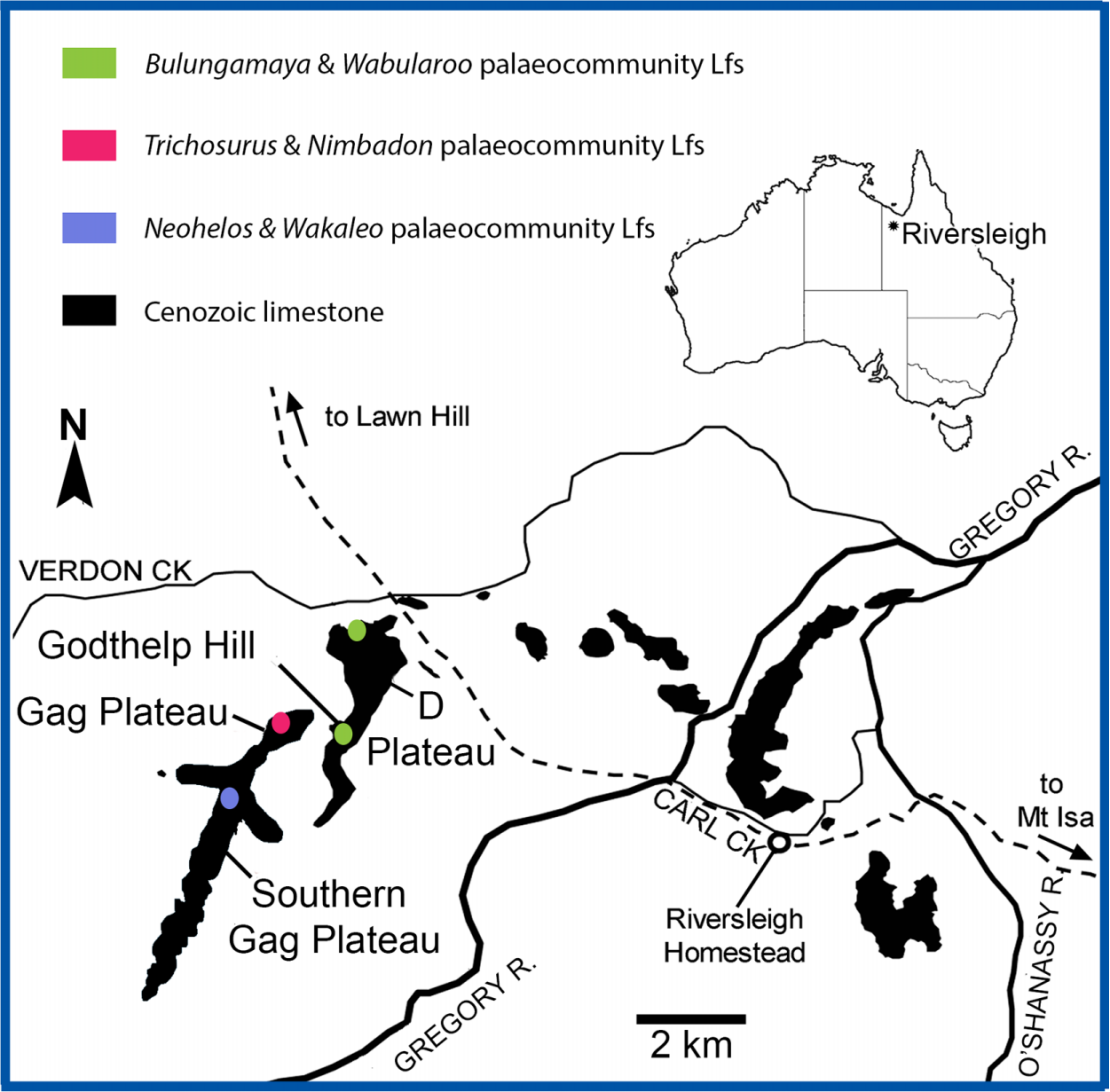
Location of the Riversleigh World Heritage Area, Queensland, Australia and local faunas representing proposed palaeocommunities (after Arena, 2004; Black, 2016).

Over 200 fossil localities spanning the late Oligocene to late Pleistocene have been identified from the Riversleigh World Heritage Area in north-western Queensland (Fig. 1; Archer et al., 1989; Archer, Hand & Godthelp, 1995). Each site is characterised by a constituent fossil assemblage, lithology and taphonomic history. Traditionally, the biota of each site has been designated a local fauna, defined as the assemblage of animals collected from one locality (Tedford, 1970; Archer et al., 1989), with the expectation that future investigations would allow for aggregation of these local faunas into faunas (*sensu* Tedford, 1970). Riversleigh’s Oligo-Miocene assemblages are currently segregated into four Faunal Zones (FZ A–D) based on stratigraphy and stage of evolution biocorrelation: (Archer et al., 1989; Archer, Hand & Godthelp, 1995; Archer et al., 1997; Black, 1997a, 1997b; Myers & Archer, 1997; Myers et al., 2001; Arena, 2004; Travouillon et al., 2006; Black, 2010, 2013; Arena et al., 2015) Faunal Zone A spans the late Oligocene, FZ B the early Miocene and FZ C the middle Miocene, while FZ D possibly represents the start of the late Miocene. Ongoing radiometric (U-Pb) dating of speleothems has provided absolute dates for ten Riversleigh assemblages to date (Woodhead et al., 2011, 2016).

Archer et al. (2006) compiled current species lists for all Riversleigh sites, Travouillon et al. (2009) examined individual local faunas for determination of palaeohabitats, while Travouillon et al. (2006) compared Riversleigh local faunas using multivariate techniques for determination of relative age relationships within the Riversleigh fossil-field and with other faunas. Other studies concerning the spatial and temporal synecology of Riversleigh fossil faunas include observations on the autecology of individual species and a study of Peramelemorphian palaeoguilds (J. Muirhead, 1996, unpublished data). However, the essential determination of Riversleigh’s palaeocommunities remained to be undertaken.

Palaeocommunities are the primary operational unit for analyses of palaeoenvironmental gradients, diversity and ecomorphology on a regional to global scale. Palaeocommunities are replete with collective biological information that is unlikely to be available to researchers working solely with individual assemblages encompassing potentially significant taphonomic and sampling biases.

Herein, a *palaeocommunity* equates to the aggregate of local faunas (‘local palaeocommunities’ *sensu* Bennington & Bambach, 1996) that cannot be differentiated statistically, while the palaeocommunity *type* is the grouping of local faunas and palaeocommunities that occur in stratigraphically similar settings, can be identified using cluster analysis and are ‘similar, but statistically significantly different’ (Bennington & Bambach, 1996, p. 112). The determination of palaeocommunities is fundamental to palaeoecology; indeed Etter (1999) suggests that this is the ultimate goal of palaeoecological classification.

In this study, we use classification and ordination techniques, now well established as complementary (Bennington & Bambach, 1996; Brenchley & Harper, 1998; Digby & Kempton, 1987; Etter, 1999; Hammer & Harper, 2006), to examine a suite of sites, including some of the best-sampled Riversleigh local faunas, to determine those that cannot be palaeoecologically differentiated and should therefore be considered to be palaeocommunities, as well as clusters of similar palaeocommunities that represent palaeocommunity types.

For the first time Riversleigh’s Oligo-Miocene palaeocommunities (and associated types) are formalised, allowing future research to analyse more informative taxonomic datasets rather than relying solely on data from individual local faunas. Riversleigh fossil assemblages provide a rare opportunity to study the synecology of a palaeocommunity (or palaeocommunities) changing over a significant period of geological time, through cycles of climatic change and associated extinction events.

Principally the aims of this study are to determine:

1. If palaeocommunities can be identified among Riversleigh’s better-sampled local faunas;
2. The relationship, if any, of identified palaeocommunities to established Riversleigh Faunal Zones;
3. If taxonomic level affects identification of palaeocommunities;
4. How changes in palaeocommunity structure may relate to palaeoclimate;
5. The utility of recognised clustering and ordination techniques, as well as similarity/dissimilarity indices, in palaeocommunity recognition.

## Methods

### Choice of sampling units

Fourteen local faunas (Lfs) representing ‘snapshots’ in time over the Oligo-Miocene (FZ A–D) temporal range of Riversleigh’s fossil deposits, were analysed (Table 1). Faunal Zone A (late Oligocene) Lfs include: hiatus (HI); and white hunter (WH). Faunal Zone B (early Miocene) Lfs include: camel sputum (CS); Mike’s menagerie (MM); Neville’s garden (NG); upper (UP); and Wayne’s wok (WW). Faunal Zone C Lfs include: Cleft-of-ages (COA); Gag (GAG; Archer & Hand (1984) named this the Dwornamor Lf but for ease of use it is referred to as the GAG Lf in this analysis); Henk’s hollow (HH); Keith’s chocky block (KCB); last minute (LM); and ringtail (RING). Faunal Zone D Lfs include: Encore (ENC). Absolute radiometric dates have so far been determined for three of the sampled sites (∼18 Ma for NG and CS, and ∼13.5 Ma for RING; Woodhead et al., 2016). Each local fauna within the study sample was chosen for its uniformity of lithology, lack of stratification, the degree of horizontal and vertical confinement of each site, as well as by the sampling effort already undertaken.

**Table 1:** Local fauna characteristics.

Local fauna	Abbreviation	Biochron^1^	Age^2^	Palaeocommunity
White hunter	WH	A	Late Oligocene (28.1–23.03 Ma)	–
Hiatus	HI	A–C	Late Oligocene? (28.1–23.03 Ma)	–
Wayne’s wok	WW	B3	Early Miocene (23.03–15.97 Ma)	*Bulungamaya & Wabularoo*
Camel sputum	CS	B3	Early Miocene (18.53–16.97 Ma)*	*Bulungamaya & Wabularoo*
Mike’s menagerie	MM	B3	Early Miocene (23.03–15.97 Ma)	*Bulungamaya & Wabularoo*
Neville’s garden	NG	B3	Early Miocene (17.95–17.98 Ma)*	*Bulungamaya & Wabularoo*
Upper	UP	B3	Early Miocene (23.03–15.97 Ma)	*Bulungamaya & Wabularoo*
Gag (Dwornamor)	GAG	C2–C3	Middle Miocene (15.97–11.63 Ma)	*Trichosurus & Nimbadon*
Henk’s hollow	HH	C2	Middle Miocene (15.97–11.63 Ma)	*Trichosurus & Nimbadon*
Last minute	LM	C1–C3	Middle Miocene (15.97–11.63 Ma)	*Trichosurus & Nimbadon*
Ringtail	RING	C1–D	Middle Miocene (14.23–12.89 Ma)*	*Trichosurus & Nimbadon*
Cleft-of-ages	COA	C1–C3	Middle Miocene (15.97–11.63 Ma)	*Neohelos & Wakaleo*
Keith’s chocky block	KCB	C1	Middle Miocene (15.97–11.63 Ma)	*Neohelos & Wakaleo*
Encore	ENC	D	Early late Miocene? (11.63–7.25 Ma)	–

**Notes:**

1 Arena et al. (2015).

2 Cohen et al. (2013), updated.

* Absolute date from Woodhead et al. (2016).

### Datasets and counting methods

Datasets of taxonomic presence/absence and relative abundance were compiled from a combination of sources, including the Riversleigh Project specimen database (30,000+ specimens registered in the Vertebrate Palaeontology Laboratory, University of New South Wales and the Queensland Museum) and published papers (Archer et al., 2006; Travouillon et al., 2011). More than 15,000 fossils (including individual and bulk specimens) were examined to confirm counts and previous published and unpublished taxonomic identifications. Raw abundance data were recorded as the number of identified specimens (NISP) at superfamily (Table S1), family (Table S2), genus (Table S3) and species (Table S4) level. Presence/absence and NISP data were intermittently updated over a 20 year period (1996–2016) to allow for taxonomic revisions. Nevertheless, given that taxonomy of Riversleigh specimens is in a constant state of flux and the wealth of new material being discovered and prepared each year, these NISP values should be considered minimum values.

Number of identified specimens values were used in preference to other counting methods, such as the minimum number of individuals (MNI), following Grayson (1984) who identified that: (1) NISP is more convenient to use when dealing with a large quantity of bone material because it is not necessary to associate skeletal elements, only to determine their taxonomy; (2) MNI is a function of NISP, so that any perceived problems with using NISP (such as association of elements) will also affect MNI determination; and (3) MNI is heavily influenced by the way in which the investigator arbitrarily divides their fossil sites into stratigraphic units.

**Figure 2:**
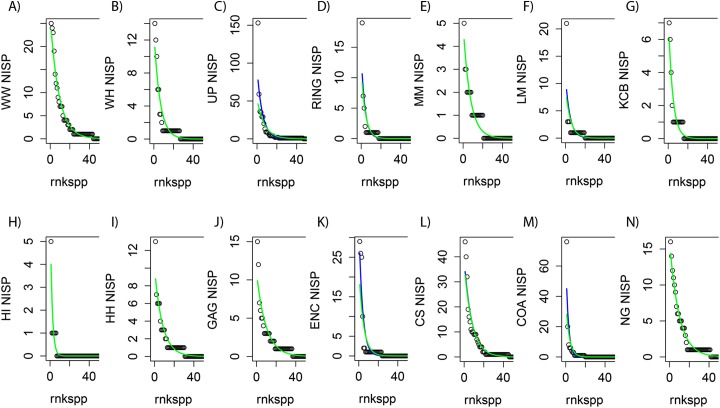
Species-level NISP generalised linear models (GLMs) for Riversleigh local faunas (Lfs). rnkspp = rank order of species. Green line is negative binomial (NB) response. Blue line is Poisson (POIS) response. For nine Lfs (A, B, E, G, H, I, J, L and N) the responses do not differ, with NB not fitting better than POIS, as determined by likelihood ratio test (*p* ≫ 0.05; green line overlying blue line). For RING Lf (D) the responses diverge, but not significantly, so POIS is preferred (*p* = 0.077). For LM, COA, UP and ENC Lfs (F, M, C and K) the NB distribution fits slightly better than POIS (*p* < 0.05).

When using abundance data in multivariate analyses, Etter (1999) recommends transforming values when particular species are very common, effectively giving less weight to common species and more to those that are rarer. Logarithmic transformation is generally used when some species are particularly dominant, reducing the differences between species abundances while maintaining differences in population sizes between sampling units (Etter, 1999). More recently, however, data transformation has been found to perform poorly (O’Hara & Kotze, 2010). Consequently, generalised linear models (GLMs) were applied using the raw NISP distributions as predictors, at each taxonomic level, with the MASS, LATTICE and PSCL packages for R in RStudio (Fig. 2; Venables & Ripley, 2002; Sarkar, 2008; Zeileis, Kleiber & Jackman, 2008; R Core Team, 2014; Jackman, 2015; RStudio Team, 2015). The Vuong test was used to compare the fit of Poisson (POIS) and negative binomial (NB) GLMs with their zero-inflated non-nested counterparts, while the Log-likelihood Ratio test was used to distinguish *between* POIS and NB GLMs (Vuong, 1989; Cameron & Trivedi, 1998). Raw NISP values were then adjusted for all taxa in all Lfs using response values as determined from the best-fitting GLM.

A total of 129 species, 79 genera, 31 families and 16 ‘super-families’ were included in the taxonomic datasets analysed (taxonomy follows: Archer et al., 2006; Travouillon et al., 2011; Black, Archer & Hand, 2012; Black et al., 2012; Tables S1–S4). Families that are not formally assigned to a super-family in current classifications were, for the purpose of this analysis, designated a ‘super-familial’ grouping. For example, four families (Ilariidae, Maradidae, Wynyardiidae, Thylacoleonidae) within the Infraorder Vombatomorphia are not currently assigned to superfamilies and are grouped here as Vombatomorphia *incertae sedis* in the super-familial dataset. Taxonomic identifications are likely to be more reliable at lower taxonomic levels, due to the fact that most species and genus level assignments are made by the relevant taxonomic specialists, although the substantially larger sample sizes at higher taxonomic levels potentially reduces incorrect assignments.

### Classification/ordination and choice of similarity/dissimilarity indices

The non-volant mammalian component of each Lf was subjected to classification and ordination analyses using PAST software (version 3.08, Hammer, Harper & Ryan, 2001). Classification is recommended as the first step in any analysis of palaeocommunities because it simplifies large datasets, allows for the grouping of recurrent assemblages, and ultimately identifies palaeocommunities (Etter, 1999). Classification was performed via Q-mode cluster analysis using nine similarity and distance indices (Tables 2–5). Distance measures used for abundance data included the Chord, Bray–Curtis, Cosine, Morisita and Horn indices. Presence/absence data were examined using the Dice, Simpson and Raup–Crick indices. A ‘corrected’ Forbes index was also used due to its purported superior accuracy and resistance to sample-size effects (Alroy, 2015a). The Jaccard, Ochiai and Kulczynski coefficients were not used due to their high correlation with the Dice index (Alroy, 2015a). The ‘unweighted pair group method using arithmetic averages’ (UPGMA) and neighbour-joining clustering algorithms were used with each of the indices (Saitou & Nei, 1987; Popov, Gerasimov & Marinska, 1994; Brenchley & Harper, 1998; Etter, 1999; Hammer, Harper & Ryan, 2001). Ward’s clustering method was not employed as it has been found to be sensitive to outliers and more suitable for morphometric data (Hammer & Harper, 2006). Correlation techniques were also not utilised due to an inability to accurately reflect differences in taxonomic abundance, as well as their susceptibility to be affected by sample size and missing data (Etter, 1999; Krebs, 1989; Wolda, 1981). UPGMA and neighbour-joining results were used instead of single linkage due to problems such as ‘chaining’ common to the latter (Etter, 1999).

**Table 2:** Summary of species-level classification.

Data	Clustering algorithm	Similarity/dissimilarity index	Recurring clusters										Cophenetic correlation
			WH HI	NG UP CS WW	NG UP CS WW MM	NG UP CS WW MM WH	COA KCB	GAG HH	LM RING	GAG HH LM	GAG LM RING	GAG HH LM RING	
Presence/absence	UPGMA	Raup–Crick	✓53	–	✓81	–	✓66	✓	✓	✓	–	✓70	0.85
		Simpson	✓42	–	✓48	–	–	✓	–	✓	–	✓52	0.77
		Dice	–	✓	✓88	✓51	✓61	✓	–	✓64	–	✓58	0.93
		Forbes	–	✓	✓	✓	✓	–	–	✓	–	✓	0.85
	Neighbour joining	Raup–Crick	✓52	–	–	–	✓60	✓	–	✓34	–	–	–
		Simpson	✓61	–	–	–	✓33	✓	–	✓30	–	✓39	–
		Dice	✓43	–	✓65	–	✓60	✓46	–	–	–	–	–
		Forbes	✓	–	–	–	✓	✓	–	✓	–	✓	–
Abundance	UPGMA	Horn	✓57	✓61	✓70	–	✓81	✓	–	✓52	–	✓44	0.86
		Morisita	✓48	✓68	✓41	–	✓72	✓57	✓26	–	–	✓8	0.85
		Cosine	✓54	✓68	✓40	–	✓77	–	–	✓27	–	✓8	0.85
		Chord	✓54	✓68	✓42	–	✓77	–	–	✓30	–	✓5	0.88
		Bray–Curtis	–	✓97	–	–	–	✓	✓	✓	–	✓53	0.91
	Neighbour joining	Horn	✓66	–	✓67	–	✓87	–	–	✓44	–	–	–
		Morisita	✓60	–	✓45	–	✓82	✓63	✓41	–	–	–	–
		Cosine	✓64	–	✓47	–	✓84	✓50	✓36	–	–	–	–
		Chord	✓65	–	✓42	–	✓80	✓50	✓33	–	–	–	–
		Bray–Curtis	✓40	✓99	–	–	–	–	✓	–	✓	✓53	–

**Note:**

Ticks designate presence of cluster. Bootstrap percentage presented where available.

**Table 3:** Summary of genus-level classification.

Data	Clustering algorithm	Similarity/dissimilarity index	Recurring clusters										Cophenetic correlation
			WH HI	NG UP CS WW	NG UP CS WW MM	NG UP CS WW MM WH	COA KCB	GAG HH	LM RING	GAG HH LM	GAG LM RING	GAG HH LM RING	
Presence/absence	UPGMA	Raup–Crick	✓36	–	✓29	–	–	✓	✓	–	–	–	0.85
		Simpson	–	–	–	–	–	–	–	–	–	–	0.77
		Dice	–	✓84	–	–	✓45	✓88	✓70	–	–	–	0.93
		Forbes	–	–	–	–	–	–	–	–	–	–	0.85
	Neighbour joining	Raup–Crick	✓39	–	–	–	–	–	–	–	–	–	–
		Simpson	✓41	–	–	–	–	–	–	–	–	–	–
		Dice	–	–	✓14	–	✓47	–	✓	–	–	✓29	–
		Forbes	✓	–	–	–	–	–	–	–	–	–	–
Abundance	UPGMA	Horn	✓39	✓79	✓42	–	✓69	–	–	–	✓38	–	0.86
		Morisita	✓35	✓58	✓14	–	✓84	–	–	–	✓	–	0.85
		Cosine	✓38	✓57	✓15	–	✓84	–	–	–	✓	–	0.85
		Chord	✓41	✓65	✓16	–	✓84	–	–	–	✓	–	0.88
		Bray–Curtis	–	✓84	–	–	–	–	–	–	✓48	–	0.91
	Neighbour joining	Horn	✓58	–	–	–	✓74	–	–	–	✓51	–	–
		Morisita	✓59	✓32	–	–	✓82	–	–	–	✓31	–	–
		Cosine	✓58	✓28	–	–	✓83	–	–	–	✓43	–	–
		Chord	✓58	✓41	–	–	✓83	–	–	–	✓42	–	–
		Bray–Curtis	–	✓90	–	–	–	–	–	–	✓64	–	–

**Note:**

Ticks designate presence of cluster. Bootstrap percentage presented where available.

**Table 4:** Summary of familial classification.

Data	Clustering algorithm	Similarity/dissimilarity index	Recurring clusters										Cophenetic correlation
			WH HI	NG UP CS WW	NG UP CS WW MM	NG UP CS WW MM WH	COA KCB	GAG HH	LM RING	GAG HH LM	GAG LM RING	GAG HH LM RING	
Presence/absence	UPGMA	Raup–Crick	–	–	–	–	✓	–	–	–	–	–	0.85
		Simpson	–	–	–	–	–	–	–	–	–	–	0.77
		Dice	–	–	–	–	✓47	–	–	–	–	–	0.93
		Forbes	–	–	–	–	–	–	–	–	–	–	0.85
	Neighbour joining	Raup–Crick	–	–	–	–	–	–	–	–	–	–	–
		Simpson	–	–	–	–	–	–	–	✓	–	–	–
		Dice	–	✓34	–	–	–	–	✓41	–	–	–	–
		Forbes	–	–	–	–	–	–	–	–	–	–	–
Abundance	UPGMA	Horn	–	✓30	–	–	–	–	–	–	–	–	0.86
		Morisita	–	–	–	–	✓65	–	–	–	–	–	0.85
		Cosine	–	–	–	–	✓73	–	–	–	–	–	0.85
		Chord	–	–	–	–	✓72	–	–	–	–	–	0.88
		Bray–Curtis	–	✓34	–	–	–	–	–	–	✓20	–	0.91
	Neighbour joining	Horn	–	–	–	–	–	–	–	–	✓25	–	–
		Morisita	–	–	–	–	✓52	–	✓45	–	–	–	–
		Cosine	–	–	–	–	✓52	–	–	–	✓34	–	–
		Chord	–	–	–	–	✓64	–	–	–	–	–	–
		Bray–Curtis	–	✓63	–	–	–	–	–	–	✓47	–	–

**Note:**

Ticks designate presence of cluster. Bootstrap percentage presented where available.

**Table 5:** Summary of ‘super-familial’ classification.

Data	Clustering algorithm	Similarity/dissimilarity index	Recurring clusters										Cophenetic correlation
			WH HI	NG UP CS WW	NG UP CS WW MM	NG UP CS WW MM WH	COA KCB	GAG HH	LM RING	GAG HH LM	GAG LM RING	GAG HH LM RING	
Presence/absence	UPGMA	Raup–Crick	–	–	–	–	–	✓25	–	–	–	–	0.85
		Simpson	–	–	–	–	✓	–	–	–	–	–	0.77
		Dice	–	–	–	–	–	✓30	–	–	–	–	0.93
		Forbes	–	–	–	–	–	–	–	–	–	–	0.85
	Neighbour joining	Raup–Crick	–	–	–	–	–	✓11	–	–	–	–	–
		Simpson	–	–	–	–	–	–	–	–	–	–	–
		Dice	–	–	–	–	–	–	–	–	–	–	–
		Forbes	–	–	–	–	–	–	–	–	–	–	–
Abundance	UPGMA	Horn	–	–	–	–	–	–	–	–	–	–	0.86
		Morisita	–	–	–	–	–	–	–	–	–	–	0.85
		Cosine	–	–	–	–	–	✓33	–	–	–	–	0.85
		Chord	–	–	–	–	–	✓36	–	–	–	–	0.88
		Bray–Curtis	–	–	–	–	–	–	–	–	✓21	–	0.91
	Neighbour joining	Horn	–	–	–	–	–	–	–	–	–	–	–
		Morisita	–	–	–	–	–	–	✓36	–	–	–	–
		Cosine	–	–	–	–	–	–	✓39	–	–	–	–
		Chord	–	–	–	–	–	–	✓38	–	–	–	–
		Bray–Curtis	–	–	–	–	–	–	–	–	✓31	–	–

**Note:**

Ticks designate presence of cluster. Bootstrap percentage presented where available.

### Determination of palaeocommunities

All clustering results were examined to find commonly reoccurring clusters of Lfs as the primary step in any determination of palaeocommunities. Recurring clusters were considered potential palaeocommunities or palaeocommunity *types*, to be examined further through ordination (following Etter, 1999). ‘Cut-off’ levels of similarity/distance used to delimit palaeocommunities and *types* were defined for each index and algorithm combination (Table 6).

**Table 6:** Species-level similarity/distance cut-off limits for palaeocommunities and palaeocommunity *types*.

Data	Similarity/distance index	Palaeocommunity cut-off level	Palaeocommunity *type* cut-off level
Presence/absence	Simpson	0.6	–
	Dice	0.4	0.36
	Raup–Crick	0.95	–
	Forbes	−0.4	−0.5
Abundance	Horn	0.42	–
	Morisita	0.42	0.3
	Cosine	0.36	0.35
	Chord	1.13	1.14
	Bray–Curtis	0.3	–

Ordination techniques, used herein to confirm and further explain classification results (following, e.g. Legendre & Legendre, 1984), included principal components analysis (PCA), principal co-ordinates analysis (PCO) and non-metric dimensional scaling (NMDS) for presence/absence and abundance datasets, and detrended correspondence analysis (DCA) for abundance data alone (following, e.g. Etter, 1999; Hammer & Harper, 2006; Hammer, Harper & Ryan, 2001). Alroy (2015b) postulates that NMDS and PCO generally outperform DCA, while the latter produces more reliable results than PCA. These conclusions are examined herein.

ANOVA was used to compare median cophenetic correlations *between* and *within* all presence/absence and abundance indices at each taxonomic level. PERMANOVA tests (Anderson, 2001) were used to test statistical significance between potential palaeocommunities identified by classification and re-confirmed through ordination. Dice and Horn indices were used for analysing presence/absence and abundance data respectively, with 9,999 permutations for each PERMANOVA.

## Results

### Choice of GLMs for NISP distributions

In general, GLMs utilising raw NISP values for each Lf as predictors were found to be good proxies for raw abundance distributions regardless of taxonomic level (e.g. Fig. 2). However zero-inflated models could not be fitted to all raw NISP distributions (e.g. species-level NISP for WW Lf). Where zero-inflated models were produced, Vuong tests suggested non-nested counterparts (i.e. NB or POIS GLMs) produced a better fit or were statistically identical. In addition, visual examination of GLMs, in combination with Likelihood Ratio tests, strongly suggested POIS distributions were as good or a better fit than NB distributions with the latter fitting marginally better than POIS responses in only four of 14 Lfs (e.g. Fig. 2). Given this combination of factors it was clear that POIS distributions should be used as the preferred GLM at all taxonomic levels. New NISP abundance distributions derived from POIS GLM responses were therefore used as datasets for all analyses requiring abundance data.

### Classification

**Figure 3:**
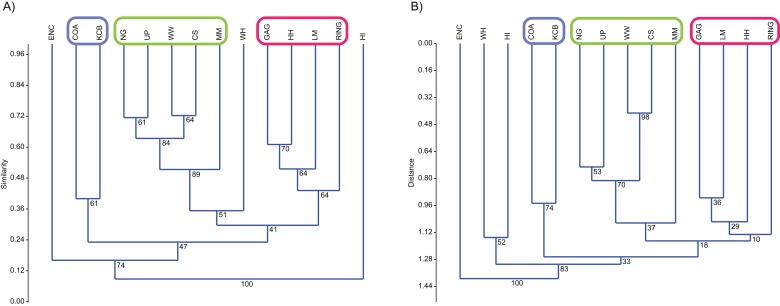
Example of species cluster analyses showing repeating clusters of palaeocommunities (blue = *Neohelos* & *Wakaleo* palaeocommunity; green = *Bulungamaya* & *Wabularoo* palaeocommunity; pink = *Trichosurus* & *Nimbadon* palaeocommunity). (A) Spp. presence/absence using UPGMA, Dice index (cophenetic correlation coefficient (ccc) = 0.93) and (B) spp. abundance using UPGMA, Chord index (ccc = 0.88). Bootstrap results at nodes.

Classification, interpreted via Q-mode cluster analysis, identified recurring groups of sampling units or Lfs (Fig. 3; Tables 2–6). These results indicate the presence of three palaeocommunities and at least one palaeocommunity *type* (*sensu* Bennington & Bambach, 1996). A cluster of NG, CS, WW and UP Lfs, indicating one natural palaeocommunity, recurs in: (1) all specific analyses utilising abundance data; (2) all analyses incorporating presence/absence data and the UPGMA algorithm; (3) a presence/absence neighbour-joining analysis using the Dice index; (4) 67% of generic analyses and (5) 22% of familial analyses (see Tables 2–5). MM Lf, one of the least-well sampled Lfs (Tables S1–S4), is also associated with the former cluster in the majority of these species-level analyses, as well as some of the generic analyses mentioned (Tables 2 and 3), suggesting that it is also a member of this palaeocommunity. Bootstrap results provide relatively strong support for the NG/WW/CS/UP cluster at the species-level, ranging from 42% (neighbour-joining Chord) to 99% (neighbour-joining Bray–Curtis; Table 2). With the addition of the under-sampled MM Lf bootstrap support decreases marginally, ranging from 40% (UPGMA Cosine) to 88% (UPGMA Dice; Table 2).

A combination of GAG, HH, LM and RING Lfs constitutes another natural grouping, being consistently linked at the species level in all analyses using the UPGMA algorithm and 33% of neighbour-joining analyses (Table 2). This cluster was recovered less often at higher taxonomic levels although combinations of two or three constituent Lfs appeared in numerous analyses (e.g. LM/RING/GAG in all generic abundance analyses and variable pair combinations at higher taxonomic levels; Tables 3–5). Bootstrap results for the GAG/HH/LM/RING cluster using species-level presence/absence or abundance data are generally not as strong as those for the NG/MM/CS/WW/UP group, ranging from 5% to 70% (Table 2). However, the lower bootstrap results appear primarily to be the result of RING Lf alone, generally linking at the base of this cluster with small bootstrap values. Excluding RING Lf from this cluster produces substantially higher median bootstrap figures, now ranging from 27% to 70% (Table 2). The fact that presence/absence bootstrap results are so much greater than those including abundance data (39–70% as against 5–53%; Table 2) suggests that the lower sample size for RING Lf is primarily responsible for the discrepancy, rather than uncertain membership of the cluster.

Constituent Lfs of the two groupings were also united in a significant number of familial analyses with combinations of Lfs from the NG/MM/WW/CS/UP and GAG/HH/LM/RING groups appearing in 66% of UPGMA and neighbour-joining analyses respectively (Table 4). Combinations of Lfs from the NG/MM/WW/CS/UP group are much less common in ‘super-familial’ analyses (appearing in only 39%), although combinations of Lfs from the GAG/HH/LM/RING cluster are present in most analyses at this level (appearing in 72%; Table 5).

Classification results also suggest that KCB and COA Lfs should form a palaeocommunity with this grouping appearing in 94% and 89% of species-level presence/absence and abundance analyses respectively (Table 2). Bootstrap results for this group range from 33% to 66% for species-level presence/absence data and are significantly higher for abundance data, ranging from 72% to 87% (Table 2). This cluster also reoccurs in numerous generic and familial analyses and more frequently in analyses using abundance data, but only occurred in one ‘super-familial’ level classification (Tables 3–5). Levels of index similarity for the KCB/COA cluster are always lower than for the NG/WW/CS/UP/MM group and the majority are also lower than for the GAG/HH/LM/RING cluster (e.g. Fig. 3A).

The classification of the WH Lf is ambiguous. In most specific and generic analyses WH and HI Lfs group together with reasonable bootstrap support, ranging from 40% to 60% (Tables 2 and 3). On its own this would seem to indicate the presence of a fourth palaeocommunity, but the consistently very low levels of index similarity indicates that it may represent instead a palaeocommunity *type* (e.g. Fig. 3B). Furthermore, in species-level analyses, utilising UPGMA with the Dice and Forbes indices, WH Lf unites with the NG/WW/CS/UP/MM grouping at higher levels of similarity than the WH/HI pairing, suggesting an alternative palaeocommunity *type* (e.g. Fig. 3A). Levels of similarity/distance for all indices are consistently higher/lower for proposed palaeocommunities relative to the NG/WW/CS/UP/MM/WH palaeocommunity type (Table 6).

Cophenetic correlation coefficients (ccc) produced for the species-level UPGMA indices were generally very high (median ccc = 0.85 for presence/absence data and ccc = 0.86 for abundance data; Table 2). At higher taxonomic levels the median ccc reduces for presence/absence data (generic ccc = 0.74; familial ccc = 0.64 and super-familial ccc = 0.73; Tables 3–5), although ANOVA suggests the difference is not statistically significant (*p* = 0.28). Similarly, ANOVA indicates no difference between presence/absence and abundance ccc medians at any taxonomic level. Only between specific (ccc = 0.86) and familial (ccc = 0.76) abundance medians was statistical significance observed (ANOVA *p* = 0.001), indicating a reduction in classification reliability using abundance data between these taxonomic levels.

For presence/absence data the Dice index had the highest ccc at all taxonomic levels, ranging from 0.8 to 0.93 (Tables 2–5). Comparison of this index with the second highest ccc for all taxonomic levels suggested a significant difference (ANOVA *p* = 0.04).

Conversely, no statistically significant difference could be found for comparison of highest and lowest ccc for all abundance indices, at all taxonomic levels (ANOVA *p* = 0.1).

For specific abundance data the Bray–Curtis index alone failed to identify the NG/WW/CS/UP/MM group (Table 2). Few algorithm-index combinations recovered this group using specific presence/absence data. Conversely, the GAG/HH/LM/RING was found more often with presence/absence data than abundance. The KCB/COA cluster was also found slightly less often with specific abundance data. Results varied at the generic level, with presence/absence data more readily identifying the NG/WW/CS/UP/MM and GAG/HH/LM/RING groups, while abundance data were more useful for the KCB/COA cluster (Table 3). For familial data only the KCB/COA group was found, revealed more frequently by abundance analyses (Table 4). The same cluster was only identified at the ‘super-familial’ level in one presence/absence analysis (Table 5).

With the exception of the Dice index similarity indices using the neighbour-joining algorithm, with specific presence/absence data, failed to recover the NG/WW/CS/UP/MM palaeocommunity (Table 2). Similarly, the GAG/HH/LM/RING was only recovered with the Forbes index when using neighbour joining. When specific abundance data are utilised, in conjunction with the neighbour-joining algorithm, all indices identified at least one palaeocommunity but none recovered all three.

With generic data the UPGMA algorithm is again more successful at revealing palaeocommunities than neighbour joining (Table 3). UPGMA of presence/absence data, at this level, identified palaeocommunities using the Dice and Raup–Crick indices. All palaeocommunities were found using neighbour joining and Dice, although none were found with any other index. When generic abundance data were used UPGMA consistently recovered more palaeocommunities than neighbour joining. At the familial level the COA/KCB group was identified with UPGMA and most indices, but only in few of the neighbour-joining indices (Table 4). Likewise, at the ‘super-familial’ level only the COA/KCB group was identified using UPGMA in conjunction with the Simpson index, while no palaeocommunities were identified using neighbour joining (Table 5).

### Ordination

#### Specific analysis

**Figure 4:**
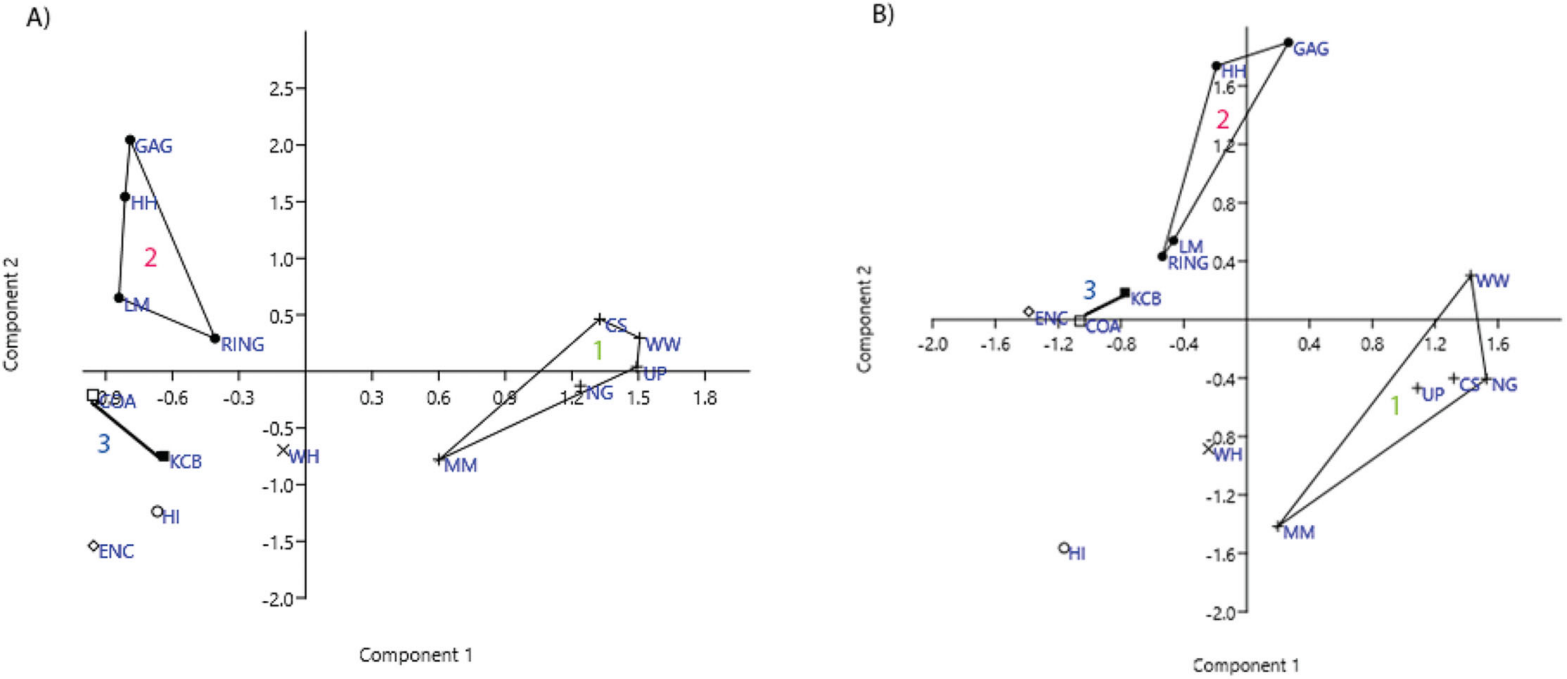
Example of species and genus ordination. Principal component analysis (PCA) of (A) spp. p/a and (B) gen. p/a. First two components represent 40% and 41% variation, respectively. Palaeocommunities represented by convex hulls and vector (‘1’ = *Bulungamaya* & *Wabularoo* palaeocommunity; ‘2’ = *Trichosurus* & *Nimbadon* palaeocommunity; ‘3’ = *Neohelos* & *Wakaleo* palaeocommunity).

**Figure 5:**
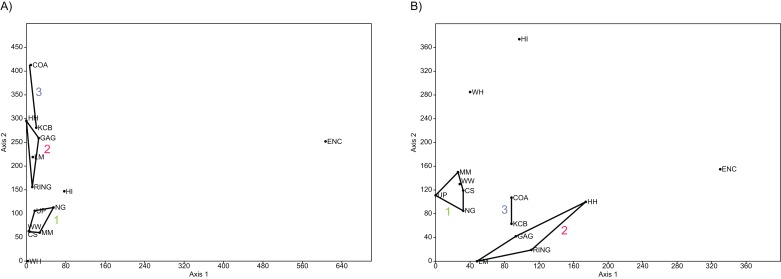
Examples of detrended correspondence analysis (DCA). (A) Species and (B) Genera. ‘1’ = *Bulungamaya & Wabularoo* palaeocommunity; ‘2’ = *Trichosurus & Nimbadon* palaeocommunity; ‘3’ = *Neohelos & Wakaleo* palaeocommunity.

**Figure 6:**
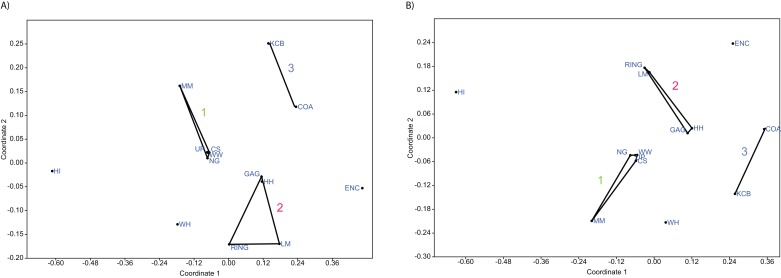
Example of non-metric multidimensional scaling (NMDS). (A) Species p/a; (B) Generic p/a. ‘1’ = *Bulungamaya & Wabularoo* palaeocommunity; ‘2’ = *Trichosurus & Nimbadon* palaeocommunity; ‘3’ = *Neohelos & Wakaleo* palaeocommunity.

**Figure 7:**
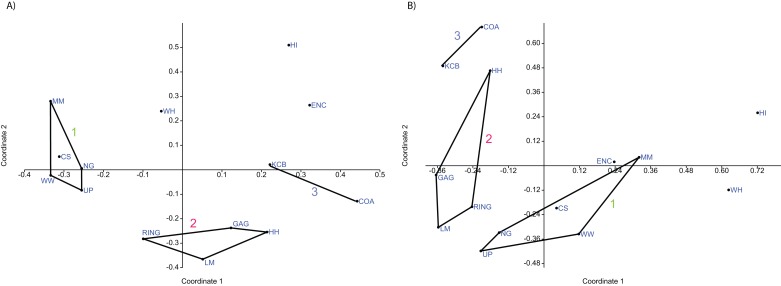
Example of principal coordinates analysis (PCO) analyses. (A) Specific abundance, Horn index; (B) Generic abundance, Chord index. ‘1’ = *Bulungamaya & Wabularoo* palaeocommunity; ‘2’ = *Trichosurus & Nimbadon* palaeocommunity; ‘3’ = *Neohelos & Wakaleo* palaeocommunity.

Ordination of presence/absence and abundance data at the specific level confirms the grouping of NG, CS, WW, UP and MM Lfs, as well as the LM, GAG, HH and RING Lfs (e.g. Figs. 4–7). Both clusters are clearly differentiated in all PCA, PCO, NMDS and DCA. Support for the KCB/COA group is less definitive, as a vector rather than a convex hull is formed from only two Lfs. The identification of other member Lfs will allow the multidimensional boundaries of this palaeocommunity to be better defined and will, ultimately, determine its validity. Nevertheless, the resultant vector does not overlap with convex hulls formed by the other groupings in any species-level PCA, PCO, DCA or NMDS. This vector is also as short as, or shorter than, the major axis of the convex hulls.

In species presence/absence PCO the WH Lf consistently occurs as the most proximal Lf to the NG/MM/WW/CS/UP cluster, strongly reinforcing the similarity of WH Lf to this group. PCA and NMDS ordination of presence/absence data is more equivocal for this palaeocommunity type with WH Lf arbitrarily appearing closest to the NG/MM/WW/CS/UP or GAG/HH/LM/RING group and occasionally midway between both (e.g. Figs. 4 and 6). Species abundance ordination also supports this palaeocommunity type, with WH Lf always being the most proximal Lf to the NG/MM/WW/CS/UP group in PCA, DCA and PCO (e.g. Figs. 5 and 7). NMDS of abundance data provides weaker support for this palaeocommunity type with WH Lf being positioned equidistance from HI Lf and the NG/MM/WW/CS/UP group, or only marginally closer to the latter.

**Figure 8:**
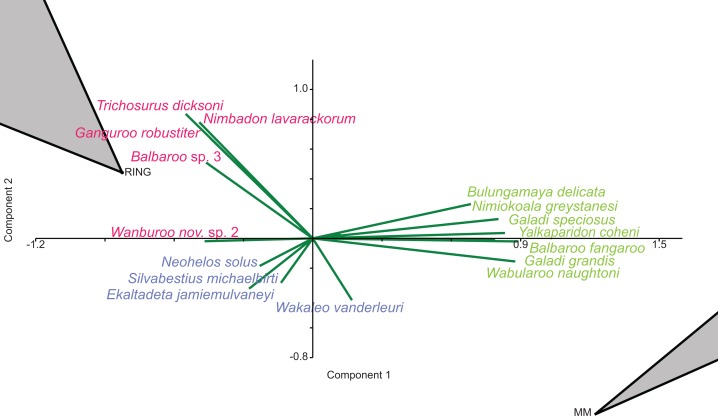
Species-level p/a PCA biplot. Green = influential taxa from *Bulungamaya & Wabularoo* palaeocommunity; Pink = influential taxa from *Trichosurus & Nimbadon* palaeocommunity; Blue = influential taxa from *Neohelos & Wakaleo* palaeocommunity. Grey polygons represent palaeocommunity partial convex hulls.

In species-level PCA using presence/absence data, the taxa with the highest positive loadings on the first principal component (PC1) include the peramelemorphians *Galadi speciosus* and *Galadi grandis*, the macropodoids *Wabularoo naughtoni*, *Balbaroo fangaroo* and *Nambaroo* nov. sp. 4, the phascolarctid *Nimiokoala greystanesi* and the enigmatic *Yalkaparidon coheni*. These species are therefore influential in forming the NG/MM/WW/CS/UP cluster, given that this group is situated on the positive side of PC1 (Fig. 4). Conversely *Trichosurus dicksoni*, *Nimbadon lavarackorum, Wanburoo* nov. sp. 2*, Ganguroo robustiter* and *Balbaroo* nov. sp. 3 exhibit high negative loadings on PC1 and are important contributors to the GAG/HH/LM/RING grouping. A potentially more informative biplot on this PCA, isolating species vectors that are directed towards hull centroids, confirms the significance of these species (Fig. 8). The biplot also identifies several species that are characteristic for the COA/KCB palaeocommunity, such as *Neohelos solus, Wakaleo vanderleuri, Ekaltadeta jamiemulvaneyi, Onirocusus rupina* and *Silvabestius michaelbirti*.

**Figure 9:**
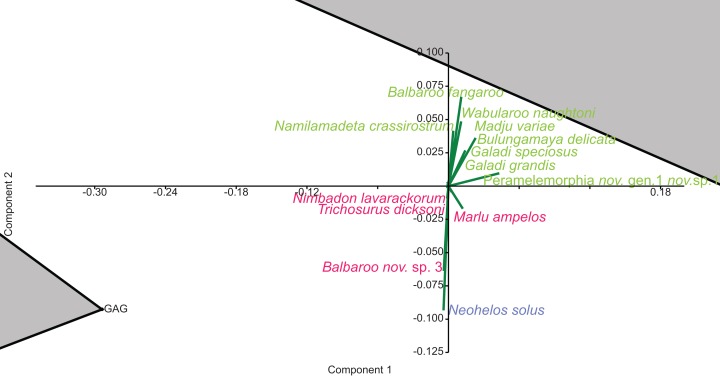
Species-level abundance PCA biplot. Green = influential taxa from *Bulungamaya & Wabularoo* palaeocommunity; Pink = influential taxa from *Trichosurus & Nimbadon* palaeocommunity; Blue = influential taxa from *Neohelos & Wakaleo* palaeocommunity. Grey polygons represent palaeocommunity partial convex hulls.

When abundance data are examined in the species PCA, via a superimposed biplot, the relative abundance of *Balbaroo fangaroo, Bulungamaya delicata, Wabularoo naughtoni, Namilamadeta crassirostrum, Galadi speciosus, Galadi grandis, Madju variae* and Peramelemorphia nov. gen. 1 nov. sp. 1 are emphasised as significant contributors to the NG/MM/WW/CS/UP grouping (Fig. 9). Equally the relative abundance of *Trichosurus dicksoni* and *Nimbadon lavarackorum*, and to a lesser extent *Marlu ampelos* and *Balbaroo* nov. sp. 3, are confirmed as important in distinguishing the GAG/HH/LM/RING cluster from other Lfs. Relatively high numbers of *Neohelos solus, Ekaltadeta jamiemulvaneyi, Wakaleo vanderleuri* and possibly *Onirocuscus rupina* and *Silvabestius michaelbirti* are also identified as important for the COA/KCB palaeocommunity.

Species-level ordination of presence/absence data does not support the similarity of WH and HI Lfs, with the relative position of each Lf varying depending on the multivariate technique or similarity coefficient employed. Likewise abundance ordination fails to highlight any particular pattern between these Lfs, with them positioned arbitrary distances from one another, the main groupings and other independent Lfs.

#### Generic analysis

Results from ordination of generic-level data are consistent with those at the specific level. Convex hulls identifying the NG/MM/WW/CS/UP and GAG/HH/LM/RING groupings are separated in both abundance and presence/absence analyses (e.g. Figs. 4–7). Strong support for the KCB/COA group is also evident, with the vector isolated from, and smaller than, the convex hulls of the other groupings in all generic presence/absence and abundance ordination.

The proposed palaeocommunity type of WH Lf and the NG/MM/WW/CS/UP group is confirmed by all generic presence/absence ordination (e.g. Figs. 4 and 6). Support is more ambiguous in generic abundance ordination because at least one other Lf is often situated closer, or equally proximal, to the NG/MM/WW/CS/UP hull than WH (e.g. Fig. 7B). Some support was found for the WH/HI palaeocommunity type at the generic level although the large size of the resultant vector combined with the proximity of WH to the NG/MM/WW/CS/UP group, particularly in presence/absence ordination, generally militates against the validity of this entity. In most analyses HI Lf was an outlier.

A biplot on the generic presence/absence PCA indicates that the presence of *Namilamadeta*, Phalangeridae nov. gen., *Wabularoo*, *Naraboryctes* and *Bulungamaya* in the NG/MM/WW/CS/UP group, as well as the presence of *Trichosurus*, *Nimbadon* and *Wanburoo* for the GAG/HH/LM/RING cluster, distinguishes the two main groupings of Lfs. Significant genera indicated on this biplot for the COA/KCB palaeocommunity are *Mayigriphus, Rhizophascolonus* and *Nimbacinus.*

For the PCA on generic abundance data, the biplot indicates that relatively high abundances of Peramelemorphia nov. gen. 3, *Yalkaparidon, Bulungamaya, Madju, Wabularoo* and to a lesser degree *Galadi* and *Naraboryctes* distinguish the NG/MM/WW/CS/UP grouping. *Trichosurus, Barinya* and *Nimbadon* are identified as important contributors to the GAG/HH/LM/RING grouping. Significant genera for determination of the KCB/COA palaeocommunity are difficult to identify but appear to include *Obdurodon*, *Wabulacinus* and *Trichosurus*.

#### Familial analysis

**Figure 10:**
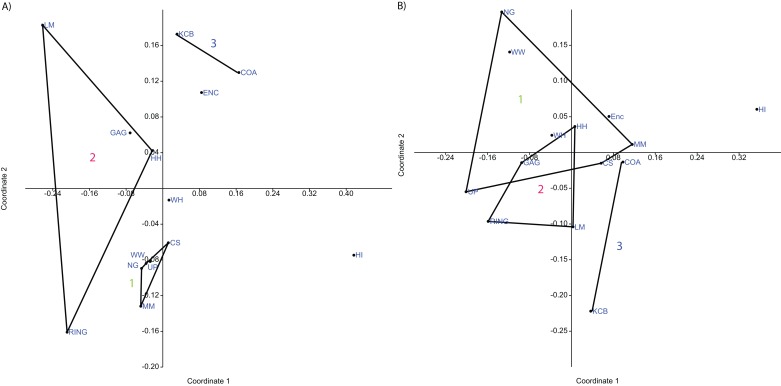
Example of familial and ‘super-familial’ ordination. (A) Familial p/a PCO, Dice index; (B) ‘Super-famillial’ p/a PCO, Dice index. ‘1’ = *Bulungamaya & Wabularoo* palaeocommunity; ‘2’ = *Trichosurus & Nimbadon* palaeocommunity; ‘3’ = *Neohelos & Wakaleo* palaeocommunity.

In familial-level presence/absence ordination, the postulated groupings, NG/MM/WW/CS/UP and GAG/HH/LM/RING, generally remain separated but are much closer than at lower taxonomic levels (e.g. Fig. 10A). Substantial overlap of the convex hulls occurred in the PCA. The KCB/COA vector is relatively short and differentiated from the two main groups in all analyses. The WH Lf occurs close to each of the main groupings, therefore not supporting a relationship with the NG/MM/WW/CS/UP group alone. The HI Lf is again situated as an outlier relative to all other Lfs, reducing support for the WH/HI palaeocommunity type.

The biplot for the familial presence/absence PCA is difficult to interpret given the size and proximity of the convex hulls for the two main groupings. Influential families are more obvious if the centroids of the hulls are compared. For instance the presence of pilkipildrids, ektopodontids, Petauroidea *incertae sedis*, and wynyardiids is significant for the NG/MM/WW/CS/UP grouping. Conversely, the presence of ornithorhynchids and, less so, pseudocheirids, acrobatids and Peramelemorphia *incertae sedis* help to distinguish the grouping of GAG/HH/LM/RING. Only the Dasyuromorphia *incertae sedis* can be identified as contributing to the KCB/COA group.

For the PCA using abundance data, the convex hull for the NG/MM/WW/CS/UP group is very large but, by focusing on the centroid and overlapping biplot, influential taxa including Peramelemorphia *incertae sedis* and yalkaparidontids, and to a lesser degree balbarids can be identified. The much smaller GAG/HH/LM/RING grouping appears to be differentiated primarily by a higher abundance of dasyurids and possibly diprotodontids. Because the vector for KCB/COA is aligned with the convex hull for the GAG/HH/LM/RING group, distinguishing families could not be determined for the former.

In general, ordination analyses of familial abundance data produce a large convex hull for the NG/MM/WW/CS/UP group. Rarely, however, is there any overlap of this hull with the much smaller GAG/HH/LM/RING hull. As was found in the presence/absence analyses, the WH Lf is not commonly associated with either of the proposed groupings in abundance ordination or with the outlying HI Lf. Similarly, there is no association between other Lfs excluded from the convex hulls.

#### Super-familial analysis

At the super-familial level there is considerable overlap between the convex hulls of the two proposed groupings in the presence/absence PCA. Comparing the position of the centroids of these hulls, in conjunction with a biplot, indicates that more taxa are present in the NG/MM/WW/CS/UP grouping relative to the GAG/HH/LM/RING grouping. Only vectors for petauroids, Peramelemorphia *incertae sedis* and to a lesser extent burramyoids and Yalkaparidontia *incertae sedis* are directed more towards the GAG/HH/LM/RING centroid, indicating greater importance. Diprotodontoids and phalangeroids are the only taxa indicated as influential for the KCB/COA vector. Up to a third of the convex hulls overlap in the presence/absence PCOs (e.g. Fig. 10B), although hulls and the KCB/COA vector are distinct in the NMDS analysis.

The WH Lf is often positioned within the NG/MM/WW/CS/UP hull, while the HI Lf is still isolated from all other Lfs. Interestingly, only the COA and ENC Lfs are consistently close, despite cluster analysis failing to resolve this combination as a possible palaeocommunity.

Ordination of super-familial abundance data again indicates some overlap between the major grouping hulls, although the centroids are still clearly separate. The WH Lf is mostly found within the NG/MM/WW/CS/UP complex. HI Lf remains isolated, while no pattern can be observed between the remaining Lfs. A PCA biplot of super-familial abundance data reveals no taxonomic vectors directed towards the centroid of either convex hull or the midpoint of the KCB/COA vector. The greater relative abundance of macropodoids and Notoryctemorphia *incertae sedis* appear to make the greatest contribution to the NG/MM/WW/CS/UP group.

PERMANOVA analyses confirmed statistical differentiation between the three proposed palaeocommunities at all taxonomic levels except for super-familial (species p/a—*F* = 5.61, *p* ≪ 0.05, species abundance—*F* = 6.40, *p* ≪ 0.05; generic p/a—*F* = 5.18, *p* ≪ 0.05, generic abundance—*F* = 5.20, *p* ≪ 0.05; familial p/a—*F* = 3.39, *p* ≪ 0.05, familial abundance—*F* = 5.22, *p* ≪ 0.05; super-familial p/a—*F* = 2.18, *p* = 0.07, super-familial abundance—*F* = 1.74, *p* = 0.20). The recurrence of GAG/HH and LM/RING pairs in some cluster analyses (Tables 2–5) hints at the possibility that these combinations represent separate palaeocommunities, rather than one larger palaeocommunity. PERMANOVA indicates that this separation cannot, however, be justified (as suggested by ordination) as the pairs are statistically indistinguishable (species p/a—*F* = 1.12, *p* = 0.33; species abundance *F* = 1.82, *p* = 0.33).

## Discussion

These results indicate that three palaeocommunities (*sensu* Bennington & Bambach, 1996) are evident in the sample of 14 local faunas analysed here: (1) a palaeocommunity comprised of WW, NG, CS, UP and MM Lfs; (2) a palaeocommunity comprised of GAG, HH, RING and LM Lfs; and (3) a palaeocommunity comprised of COA and KCB Lfs. Combinations of the macropodoid species of *Balbaroo*, *Bulungamaya* and *Wabularoo*, as well as species of the peramelemorphian *Galadi*, were indicated as important in distinguishing the NG/MM/WW/CS/UP palaeocommunity. The significance of *Wabularoo* and *Bulungamaya* was also confirmed in generic analyses, while the abundance of macropodoids was similarly identified in super-familial ordination. Because *Wabularoo* and *Bulungamaya* are presently monotypic, unlike the speciose *Balbaroo* and *Galadi*, it is proposed that the NG/MM/WW/CS/UP palaeocommunity be referred to as the *Wabularoo–Bulungamaya* palaeocommunity. Likewise, the presence and abundance of the phalangerid *Trichosurus* and diprotodontid *Nimbadon* clearly differentiate the GAG/HH/RING/LM palaeocommunity at the species and genus level. The abundance of diprotodontids was also confirmed as distinguishing the GAG/HH/LM/RING palaeocommunity. It is therefore proposed that the latter be referred to as the *Trichosurus–Nimbadon* palaeocommunity. For the COA/KCB palaeocommunity, species of the diprotodontid genus *Neohelos* and thylacoleonid genus *Wakaleo* were identified in PCA as significant, with familial abundance and super-familial presence/absence analyses confirming the importance of diprotodontids and diprotodontoids respectively. It is therefore proposed that the COA/KCB palaeocommunity be referred to as the *Neohelos–Wakaleo* palaeocommunity.

The remaining three Lfs cannot be confidently placed within either of these palaeocommunities. Nevertheless, it is likely that the complex consisting of the NG/MM/WW/CS/UP palaeocommunity and WH Lf constitutes a palaeocommunity *type* (*sensu* Bennington & Bambach, 1996), at a level higher in the palaeoecological hierarchy than the palaeocommunity. While a palaeocommunity type consisting of WH and HI Lfs cannot be discounted, supporting evidence is weaker than that for the NG/MM/WW/CS/UP and WH combination. HI and ENC Lf should remain as individual local palaeocommunities (synonymous with Lfs), pending further analysis.

### Relationship of palaeocommunities to faunal zones

The proposed palaeocommunities and independent Lfs agree with Riversleigh’s current biostratigraphic framework, namely Faunal Zones A–D (Archer et al., 1989; Archer, Hand & Godthelp, 1995; Arena, 2004; Travouillon et al., 2006). Significantly, individual deposits contributing to each palaeocommunity are both spatially and temporally related (Fig. 1). For example, the proposed NG/MM/WW/CS/UP palaeocommunity consists of Lfs derived solely from early Miocene Faunal Zone B deposits of the D-Site Plateau. Moreover, the proposed GAG/HH/LM/RING palaeocommunity consists of Lfs derived solely from middle Miocene Faunal Zone C deposits of the Gag Plateau and the proposed COA/KCB palaeocommunity from middle Miocene Faunal Zone C deposits of the southern Gag Plateau (Archer et al., 1989; Archer, Hand & Godthelp, 1995; Arena, 2004; Travouillon et al., 2006). While only three of the deposits (NG, CS, RING) in our sample have been radiometrically dated, the ages of the NG and CS Lfs overlap within uncertainty (see below), further supporting their inclusion in a single palaeocommunity (Woodhead et al., 2016). More broadly, Woodhead et al. (2016) found a clear spatial relationship between sites of similar radiometric ages: all deposits dated as early Miocene were situated on the D-Site Plateau, whereas all deposits dated as middle Miocene were located on the Gag Plateau.

The WH Lf, considered to belong to late Oligocene FZ A (Archer et al., 1989; Archer, Hand & Godthelp, 1995; Myers & Archer, 1997; Arena, 2004; Black, 2010), was not found to unite consistently with the NG/MM/WW/CS/UP (Archer et al., 1989; Archer, Hand & Godthelp, 1995; Arena, 2004) grouping in classification and was also usually separate in ordination analyses, emphasising similarity but not uniformity.

The allocation of the HI and ENC Lfs to none of the palaeocommunities or palaeocommunity type is unsurprising considering the distinctive nature of their respective faunas. The late Oligocene (FZ A) HI Lf is taxonomically poor (Tables S1–S4) and dominated by large-bodied vombatomorphian groups (see Black, 1997a, 2007, 2010), whereas the early late Miocene (FZ D) ENC Lf contains numerous derived taxa representing the first occurrence in the fossil record of genera characteristic of Pliocene assemblages (e.g., *Palorchestes*, *Warendja*, and the precursor to *Phascolarctos*) (Black, 1997b; Brewer et al., 2007; Myers et al., 2001, Black, Archer & Hand, 2012; Black, Louys & Price, 2014).

Legendre & Legendre (1984) suggest that the separation of classification clusters in ordination validate the results of classification. The palaeocommunities identified by classification are, for the most part, distinct clusters separate from the remaining Lfs in ordination analyses. Palaeocommunities are discrete clusters in all specific and generic ordination analyses and exhibit only minor overlap in some familial analyses. Overlap is more significant at super-familial level although hull centroids remain distinct. Support for the palaeocommunity *type* suggested by classification is evident in the ordination results, but not as strongly as for the palaeocommunities. The WH Lf is typically situated closest to, or within, the NG/MM/WW/CS/UP grouping at all taxonomic levels.

The first three eigenvalues should account for 40% to 80% of total variation in PCA (Etter, 1999). This is the case for all PCAs performed in this analysis, ranging from 40% for species-level presence/absence data and increasing at higher levels to a maximum of 100% for super-familial abundance data. These results reaffirm the efficacy of the PCA analyses and the reliability of the results.

Independent support for the proposed palaeocommunities is provided by a number of sources. For example, Hand & Archer (2005) suggested that similarities in bat species composition, ecomorphology and trophic structure between the early Miocene (17.11 ± 0.27 Ma; Woodhead et al., 2016) Bitesantennary Lf (not analysed here) and the nearby (<2 km) NG, UP, and WW Lfs was indicative of a single bat palaeocommunity. Similarly, after examining the distribution of the highly diverse peramelemorphian taxa from Riversleigh (J. Muirhead, 1996, unpublished data) concluded that CS and UP Lfs had numerous corresponding taxa and appeared indistinguishable. Furthermore, a preliminary cluster analysis performed on peramelemorphian species abundances suggested that NG, UP, WW, CS and MM Lfs formed a group (along with other Lfs) clearly distinct from a cluster containing HH, LM and GAG Lfs (RING Lf was not analysed). Interestingly peramelemorphians were also found to be highly influential in distinguishing groupings in the present study. Travouillon et al. (2006) also provided support for the association of some Lfs found herein, concluding that MM and CS sites were confluent and represent a Faunal Zone B (early Miocene) Lf.

Arena et al. (2015) further subdivided Faunal Zones B–C into discrete Faunal Interval Zones (B1–B3, C1–C3) on the basis of the stage-of-evolution of members of nine contemporaneous mammalian lineages including peramelemorphians, diprotodontoids, thylacoleonids and macropodoids. They found that the NG, WW, CS, UP and MM Lfs were restricted to the same Faunal Interval, B3, suggesting age equivalence of these faunas. Our analysis, based on the composition of the entire faunal assemblage, augments the findings of Arena et al. (2015) indicating these Lfs were not merely coeval but derived from a single palaeocommunity. Arena et al. (2015) were unable to resolve the GAG, HH, LM and RING Lfs to a single Interval Zone because of insufficient representation of lineage taxa within each Lf, although most Lfs were restricted to FZ C, with the exception of RING which spanned FZ C–D.

### Taxonomy and palaeocommunity recognition

The palaeocommunities and palaeocommunity type identified here are recognisable at differing taxonomic levels utilising varying datasets and differing analytical techniques. Cluster analysis of specific and generic-level presence/absence or abundance data resolves a relatively high frequency of recurring potential palaeocommunity groups with generally high levels of similarity and indicates the presence of one palaeocommunity type, although support for one of the palaeocommunities (GAG/HH/LM/RING) is slightly weaker with generic abundance data. Ordination of the same data reaffirms these results and filters out the WH/HI Lf combination that cluster analyses suggested was a *possible* palaeocommunity type.

Recognition of palaeocommunities and types is much more difficult at the familial and super-familial levels. Combinations of constituent Lfs from each of the proposed palaeocommunities are identifiable but rarely all member Lfs. Similarly, at these higher taxonomic levels ordination is able to differentiate potential groupings albeit with more overlap. Presence/absence data is generally better at resolving groups at the super-familial level, while abundance data is better for familial data.

The grouping of NG, WW, CS and UP Lfs concurs with Travouillon et al. (2006) who found that these Lfs always clustered together, while this group plus MM Lf were most often associated in cluster analyses. Similarly a combination of GAG, HH and LM Lfs was also consistently recovered. RING Lf was not found to group with LM, GAG or HH Lfs in cluster analyses, although it was closely associated with the latter in PCAs (Travouillon et al., 2006). This difference might be explained by Travouillon et al. (2006) use of specific presence/absence data alone, whereas the present analysis employed presence/absence at all taxonomic levels and in conjunction with relative abundance data. Johnson & McCormick (1999) suggest that abundance data can be more informative than presence/absence in palaeontological studies. The relatively weaker evidence for membership of MM and RING Lfs in the NG/MM/WW/CS/UP and GAG/HH/LM/RING groupings, respectively, may be further rationalised by the fact that each has lower NISP, reflecting a smaller sample size and identification effort compared to other Lfs. It is equally possible that taphonomic factors and mode of deposition may be important determinants since RING site is thought to represent a surficial pool while GAG, HH and LM are likely cave deposits (Arena, 2004; Archer, Hand & Godthelp, 1995).

### Palaeocommunities, environmental gradients and palaeoclimate

One of the most important uses of ordination is the identification of potential environmental gradients (Etter, 1999). The palaeocommunities identified here are separated primarily along the first component of the ordination which, in PCA at least, explains most variation. It is not entirely clear what palaeoenvironmental parameters are responsible for the ordering of the palaeocommunities, types and individual Lfs. However, it is likely that vegetation structure, the subject of a further study, is one potential ordering factor.

The proposed groupings of Lfs appear to be derived from relatively climatically stable time intervals. The NG/MM/WW/CS/UP group includes the NG and CS Lfs which have been dated at 18.24 ± 0.29 Ma and 17.85 ± 0.13 Ma for NG; and 17.75 ± 0.78 Ma for CS (Woodhead et al., 2016). The overlap of these temporal ranges suggests a date of approximately 18 Ma for the NG/MM/WW/CS/UP palaeocommunity as well as a relatively small depositional window. Such a date places the NG/MM/WW/CS/UP palaeocommunity prior to the onset of the Monterey carbon isotope excursion (MCIE), approaching the mid-Miocene climatic optimum (MMCO) and following a period of gradual greenhouse warming that had lasted about two million years (Holbourn et al., 2007; McGowran & Li, 1994; Zachos, Dickens & Zeebe, 2008).

Only one Lf from the GAG/HH/LM/RING palaeocommunity has been dated so far. Woodhead et al. (2016) provided an absolute date of 13.56 ± 0.67 Ma for RING Lf, suggesting that this palaeocommunity was extant one to two million years after the MMCO and subsequent rapid decline in global temperatures and expansion of the Antarctic ice expansion that characterised the completion of the MCIE (as much as a seven degree decline in sea-surface temperatures between 15 and 13 Ma; Shevenell, Kennett & Lea, 2004). However, at the time of the GAG/HH/LM/RING palaeocommunity the rate of temperature decrease had slowed significantly, possibly reversing the icehouse conditions temporarily after the *Mi3* glaciation and preceding *Mi4* (McGowran & Li, 1994; Zachos, Dickens & Zeebe, 2008). This suggests that the climate and environment had stabilised after rapid change, albeit transitory, during the period the GAG/HH/LM/RING palaeocommunity was extant. The comparative stability of the climate, combined with the discrete temporal intervals represented by the palaeocommunities, militates against the likelihood of large-scale changes in vegetation structure during the time of accumulation.

### Utility of similarity/distance measures, classification and ordination methods

At least for Riversleigh fossil faunas, it is apparent that the UPGMA algorithm is more useful than neighbour joining for identifying palaeocommunities using specific and generic presence/absence data. The Dice index proved to be the most informative for resolving potential palaeocommunities, while the Raup–Crick and Forbes indices were also useful. The Simpson index failed to recover all palaeocommunities and types identified by other methods. These results concur with Hurbalek (1982) who recommended the Dice index for presence/absence data.

Alroy (2015a, 2015b) proposed a corrected version of the Forbes coefficient, finding that this index outperformed other indices and made better allowance for variation in sample size. The results presented herein suggest that the corrected Forbes index is at least as useful, or better, than other binary similarity/dissimilarity coefficients with the exception of the Dice index. The Dice index consistently recovered proposed palaeocommunities with greater frequency at the specific and higher taxonomic levels. The superior ‘goodness of fit’ of the Dice index compared to other indices, as judged by relative ccc scores, was also determined to be statistically significant. Alroy (2015a) found that moderate to poor sampling of assemblages resulted in underestimates of similarity when using the Dice and Simpson indices. This assertion adds further support to the palaeocommunities and types found herein, given that several of the Lfs examined are clearly under-sampled (e.g. MM, HI and KCB Lfs; see Tables 2–5).

For species-level abundance data the UPGMA algorithm had better utility than neighbour joining, with only the Bray–Curtis index failing to recover all palaeocommunities. The Bray–Curtis coefficient has previously been suggested to be unsuitable for ecological samples and sensitive to sample sizes (Krebs, 1989; Hammer & Harper, 2006). This study provides some support for this assertion. Horn, Cosine, Morisita and Chord similarity indices performed equally as well as each other, as judged by ANOVA of ccc and the number of palaeocommunities recovered. The utility of the Horn, Cosine, Chord and Morisita indices, observed here, compares favourably with previous studies (Wolda, 1981; Ludwig & Reynolds, 1988; Krebs, 1989; Lesperance, 1990).

Evidence presented here is equivocal for supporting the findings of Alroy (2015a, 2015b) that NMDS and PCO ordination have greater utility than DCA and PCA. All techniques were generally very good at discerning palaeocommunities at various taxonomic levels. Lack of resolution, as indicated by the overlap of proposed palaeocommunities, is minor at the familial level and slightly more substantial at ‘super-familial’ levels in all analyses, although the majority of the hulls remained largely distinct. PCO and NMDS analyses do tend to have the WH Lf in closer proximity to the NG/WW/MM/CS/UP group. This may indicate that these techniques are slightly better for recovering weaker palaeoecological associations, such as palaeocommunity types, than PCA or DCA.

### Cautionary notes

The palaeocommunities identified here are not necessarily equivalent to neontological communities due to a number of factors, including: (1) information loss from taphonomic processes as the life assemblage, or biocoenosis, progresses to the death assemblage (thanatocoenosis) and eventually to the biased fossil assemblage (taphocoenosis); (2) time-averaging, which confuses attempts to identify contemporaneous individuals in a fossil assemblage; and (3) a lack of precise information regarding the biology of extinct species (such as species distributions) (Bennington & Bambach, 1996).

Etter (1999) notes that any sampling units are time-averaged accumulations that lasted on average several thousand years, and are possibly mixed assemblages representing different palaeocommunities. However, in any analysis of multiple assemblages it soon becomes clear that certain species always occur together and probably belonged to the same community. The recurrence of species combinations over multiple sampling units, usually with a characteristic pattern of relative abundances, is usually the measure by which palaeocommunities are recognised (Bennington & Bambach, 1996; Etter, 1999). Popov, Gerasimov & Marinska (1994) conclude that taxa which are most associated together as recurrent groups could be viewed as possibly having lived together in the past as a community. The recurrence of species at particular abundances at several sites is indicative of recurring structure in a local community, and consequently implies that the sites represent the same ecological entity (Bennington & Bambach, 1996).

Bennington & Bambach (1996) define a *local* palaeocommunity as ‘…the assemblage collectable from a single bed at one outcrop, assuming that sedimentological and taphonomic interpretation suggest that the fossil deposit is generally untransported,’ holding the same position in the palaeoecological hierarchy as local communities do in the neoecological hierarchy. The Riversleigh Lfs examined here are consistent with these criteria in that they generally possess few signs of significant transportation, are lithologically uniform and spatially confined, and exhibit no obvious anomalies suggestive of a mixed assemblage such as faunal lineages with varying ‘stages of evolution’ (*sensu* Archer & Bartholomai, 1978; Travouillon et al., 2006; Arena et al., 2015).

Bennington & Bambach (1996) suggest that the local palaeocommunity may be a more accurate representation of a living community determined through the sampling of species at a single location, because: (1) more of the total species assemblage that lived in the local habitat over time will be represented; and (2) the effects of species patchiness in living communities, are reduced in palaeocommunities because they are aggregates of living communities. Furthermore, taphonomy can be largely discounted as a factor in producing similar clusters because it is generally considered to be a disorganising process. Samples drawn from the same underlying species abundance distributions are expected to vary to some degree due to taphonomic and sampling biases. It is also extremely unlikely that taphonomic processes would generate similar fossil assemblages from dissimilar living communities (Bennington & Bambach, 1996).

## Conclusion

As a result of this analysis the following conclusions can be made:

1. Palaeocommunities can be identified among Lfs from the Riversleigh World Heritage Area;
2. NG, WW, UP, CS and MM Lfs, from Faunal Zone B, are samples of the same palaeocommunity (the *Wabularoo–Bulungamaya* palaeocommunity);
3. GAG, HH, LM and RING Lfs, from Faunal Zone C, are samples of the same palaeocommunity (the *Trichosurus–Nimbadon* palaeocommunity);
4. KCB and COA Lfs, from Faunal Zone C, are samples of the same palaeocommunity (the *Neohelos–Wakaleo* palaeocommunity);
5. The NG/WW/UP/CS/MM palaeocommunity and the WH Lf are sufficiently similar to warrant inclusion in a palaeocommunity *type*, with both entities being more similar to each other than either is to the GAG/HH/LM/RING palaeocommunity;
6. ENC and HI Lfs should be considered as independent Lfs sufficiently different from one another and the palaeocommunities identified here, unless further sampling and analysis suggests otherwise;
7. Taxonomic level does affect palaeocommunity identification with species presence/absence and abundance, as well as generic presence/absence data, having the greatest utility (palaeocommunities are rarely or partially recovered at higher taxonomic levels);
8. The NG/WW/UP/CS/MM and GAG/HH/LM/RING palaeocommunities frame the Miocene Carbon Isotope Excursion (∼18 to 13.5 Ma), reflecting a change in palaeocommunity structure as a response to significant climate change;
9. The UPGMA algorithm should be used, in conjunction with the Dice index, in future analyses on specific and/or generic presence/absence data for the determination of new palaeocommunities and types;
10. The UPGMA algorithm should be used in conjunction with the Horn, Cosine, Morisita and Chord distance coefficients, in preference to the neighbour-joining algorithm or Bray–Curtis index, when attempting to discern palaeocommunities with, primarily, specific abundance data;
11. Cluster analysis should be used first to highlight possible palaeocommunities and *types* (using species-level algorithm/index ‘cut-off’ limits for Riversleigh’s local faunas (Table 6)) before undertaking confirmation and qualification of their similarity with ordination and *post-hoc* tests such as PERMANOVA.

## Supplemental Information

10.7717/peerj.3511/supp-1 Supplemental Information 1 ‘Super-familial’ raw NISP.

10.7717/peerj.3511/supp-2 Supplemental Information 2 Familial raw NISP.

10.7717/peerj.3511/supp-3 Supplemental Information 3 Generic raw NISP.

10.7717/peerj.3511/supp-4 Supplemental Information 4 Specific raw NISP.

## Additional Information and Declarations

### Data Availability

The following information was supplied regarding data availability: The raw data has been supplied as Supplemental Dataset Files.
